# Multimodality Treatment in Ewing's Sarcoma Family Tumors of the Maxilla and Maxillary Sinus: Review of the Literature

**DOI:** 10.1155/2016/3872768

**Published:** 2016-06-16

**Authors:** David Thorn, Christoph Mamot, Fatime Krasniqi, Frank Metternich, Sven Prestin

**Affiliations:** ^1^Division of Medical Oncology, Cantonal Hospital Aarau, 5001 Aarau, Switzerland; ^2^Division of Medical Oncology, University Hospital Basel, 4031 Basel, Switzerland; ^3^Division of Ear, Nose and Throat, Head & Neck Surgery, Cantonal Hospital Aarau, 5001 Aarau, Switzerland

## Abstract

The Ewing sarcoma family of tumors (ESFT) encompasses a group of highly aggressive, morphologically similar, malignant neoplasms sharing a common spontaneous genetic translocation that affect mostly children and young adults. These predominantly characteristic, small round-cell tumors include Ewing's sarcoma of the bone and soft tissue, as well as primitive neuroectodermal tumors (PNETs) involving the bone, soft tissue, and thoracopulmonary region (Askin's tumor). Extraosseous ESFTs are extremely rare, especially in the head and neck region, where literature to date consists of sporadic case reports and very small series. We hereby present a review of the literature published on ESFTs reported in the maxilla and maxillary sinus region from 1968 to 2016.

## 1. Introduction

Since the latest WHO classification of 2013 [1], Ewing sarcoma family of tumors (ESFT) encompasses a group of highly aggressive, morphologically similar, malignant neoplasms sharing a common spontaneous genetic translocation. These predominantly characteristic, small round-blue-cell tumors include classical Ewing's sarcoma of the bone, extraosseous and soft tissue Ewing's sarcoma, as well as primitive neuroectodermal tumors (PNETs) involving the bone, soft tissue, and the chest wall, and the latter also is referred to as Askin's tumor [2]. PNETs, a group of tumors classified by their common neuroectodermal origin were formerly subdivided into three major groups: (1) central PNETs (cPNET), including tumors arising from the central nervous system, such as medulloblastoma; (2) neuroblastoma, including tumors arising from the autonomic nervous system; and (3) peripheral PNETs (pPNET) referring to PNETs arising outside the central nervous system [3]. The classification and terminology of tumors belonging to the PNET-group were however not uniform and proved awkward from early on. Although the initial description of peripheral PNETs was made in 1918 by Stout [4], who described a malignant tumor of the forearm that grew axons in tissue culture, confirming its neural origin and association with neuroblastoma, Hart and Earle [5] introduced the term PNET in 1973 to characterize medulloblastoma-like lesions found in the cerebral hemispheres. In 1979, Askin et al. published a retrospective analysis of young patients with a diagnosis of small cell tumors of the thoracopulmonary region, encompassing the years from 1964 to 1976 [2]. Those small cell tumors, that did not fit the criteria of Ewing's sarcoma, lymphoma, rhabdomyosarcoma, or neuroblastoma, were designated as malignant, small cell tumors of the thoracopulmonary region, later on known as Askin's tumors. In the 1980s, the term peripheral PNET was reestablished to describe a group of soft tissue tumors of presumed neural-crest origin that presented outside the CNS and in the sympathetic nervous system.

Ewing's sarcoma, the second most common primary bone tumor in children and adolescents, was initially described by Ewing in 1921 [6] as an undifferentiated tumor involving the diaphysis of long bones that, in contrast to osteosarcomas, was radiation sensitive. PNETs have been notoriously difficult to differentiate from Ewing's sarcoma and other ESFTs considering their close molecular biological relationship. There have actually been many cases of PNETs confused with rhabdomyosarcoma, neuroblastoma, and even lymphoma. Light microscopically, PNETs are described as small, round cells that often form characteristic lobular or pseudorosette patterns, known as the Homer-Wright rosette. Their neuroectodermal differentiation is suggested by the presence of ganglion cells and neurofibrillary structures, which reveal electron-dense neurosecretory-like granules, filaments, and microtubules under the electron microscope [7–9]. Immunohistochemical evaluation shows positive staining for neuron-specific enolase, synaptophysin, S100 protein, and MB2 monoclonal antibodies [10, 11]. Diagnosis is therefore based on ultrastructural, immunohistochemical, and molecular biological investigations. When identifying tumor cells as PNETs and differentiating between Ewing's sarcoma and other small round-cell tumors, positive staining using polyclonal or monoclonal antibodies against at least two neuroendocrine or neural markers in combination with the histological detection of Homer-Wright rosettes is regarded confirmatory. As the possibilities in distinguishing between these entities constantly improved in the 80s and 90s, it was reported that the true incidence of PNETs might be indefinitely higher than assumed in older series [12–17].

Ewing's sarcoma cells, unlike PNETs, have been generally described to fail immunohistochemical staining with antibodies against neuron-specific enolase and S-100 protein [18–21], supporting the debate against their neural-crest origin. Alongside studies that report of positive immunohistochemical staining with antibodies against intermediate filaments [22], ultrastructural examination, and failure of staining with endothelial lysozyme, alpha-1 antitrypsin, alpha-1 antichymotrypsin, and immunoglobulin markers [23, 24], this forms a strong body of evidence suggesting Ewing's sarcoma cells originate from uncommitted, primitive mesenchymal cells. Both Ewing's sarcoma and PNETs show strong expression of the cell surface glycoprotein MIC2 (CD99) [25]; although not exclusively specific for these tumors, this marker is definitely regarded characteristic of them and very useful in the differential diagnosis from other small round-cell neoplasms.

Meanwhile, cytogenetic studies have led to the identification of the nonrandom t(11;22)(q24;q12) chromosome rearrangement [26, 27] in Ewing's sarcoma, PNET, Askin's tumor, and neuroepithelioma, thereby supplying strong proof of their common histogenesis. It is upon the basis of this mutual genetic aberration, which provides a valuable characteristic for their differential diagnosis from other small round-cell tumors that these entities are now collectively recognised as Ewing's sarcoma family of tumors (ESFT).

Different fusions of the EWS gene (EWSR1) on chromosome 22q12 with various members of the ETS gene family (FLI1, ERG, ETV1, ETV4, and FEV) have been described [28, 29]. The chimaeric fusion transcript EWS–FLI1 is the result of fusion of the EWS gene on 22q12 with the FLI1 gene on 11q24. Substitution of the EWS domain with a portion of the FLI1 transcriptional domain results in an EWS–FLI1 fusion transcript with increased transcriptional activity. The EWS-FLI1 fusion transcript is found in approximately 85% of cases and considered pathognomonic [28, 30]. In the remaining 15% of tumors, other EWS-ETS gene family rearrangements have been identified, the second most common being the t(21;22)(q22;q12) translocation resulting in fusion of EWS with the ERG gene on 21q22 [31].

The EWS-ETS fusion proteins have been shown to activate human telomerase activity in Ewing's sarcoma through upregulation of TERT (telomerase reverse transcriptase) gene expression, probably by functioning as a transcriptional coactivator [32]. The oncogenic effect of EWS-ETS fusion transcripts, may be partly mediated by upregulation of LAMB3 expression, a gene encoding the *β*3 chain of laminin-5. Laminin-5 is frequently found to be strongly expressed in the cytoplasm of invading cancer cells, suggesting its role at the invasive front of colorectal, gastric, pancreatic, and breast tumors, alongside various others [33]. Many malignancies, experience loss of cell cycle control during multistage progression. In ESFTs, studies have demonstrated changes in G1/S regulatory genes after downregulation and forced expression of the EWS–FLI1 fusion gene [34], supporting the hypothesis that abrogation of the G1 checkpoint appears to be important in the progression and development of the clinical phenotype [35–37].

Although rare in adults, classical osseous Ewing's sarcoma constitutes the second most frequent primary bone cancer in children after osteosarcoma. Nevertheless, with an annual incidence of approximately 0.6/million total population, affecting 13/million 0–24 year olds each year in the UK [38], this is still a rare disease even among the adolescent population. Most commonly, patients are diagnosed with Ewing's sarcoma in the second decade of life, although 20–30% of cases are reported to occur in the first decade. The male to female ratio is approximately 1.3 : 1, so young boys are at a slightly higher risk than girls [39]. Caucasians are generally far more frequently affected than Asians, African-Americans, or Africans [40, 41]. Ewing's sarcoma usually involves the central and peripheral skeleton, namely, the pelvis and long bones, whereas involvement of nonosseous tissue is rare. ESFTs in the head and neck region are extremely rare, accounting for a mere 1%–7% of cases [39, 42, 43]. Generally, literature addressing Ewing's sarcoma family tumors in the head and neck region consists of sporadic case reports or very small series. Many of these series however fail to differentiate between the exact primary tumor locations by classifying “head and neck” as a collective potpourri [42, 44–48]. Although reported 5-year overall survival rates for Ewing's sarcoma family tumors in all sites have increased markedly thanks to modern multimodality treatment, reaching almost 70% in localized disease, only minimal data is available on the outcome of ESFTs located specifically in the head and neck region. Reports of pPNETs involving the maxilla and/or maxillary sinus are exceedingly rare; they predominantly lack immunohistochemical confirmation as well as long-term follow-up data after treatment. The following systematic review of the literature on ESFTs reported in the maxilla and maxillary sinus region aims to elucidate the background of these seldom neoplasms, detect analogies in their management, and give an overview of the modern multimodality treatment options available. We hope to offer guidance and support to clinicians facing the challenges of treating ESFTs in this confined region.

## 2. Methods

We performed a concise electronic research for reported cases of Ewing sarcoma family of tumors published in English medical literature, involving the maxilla and maxillary sinus only. The PubMed database was systematically searched for the terms Ewing's sarcoma, Ewing sarcoma family of tumors, Ewing, and PNET, in combination with the anatomical location sites:maxilla, maxillary sinus, upper jaw, and face, as well as head and neck. Single cases and case series describing ESFTs in this region were studied meticulously and the data extracted. Those cases of PNETs and Ewing's sarcomas not strictly limited to the maxilla or maxillary sinus, for instance, involving the mandible, the orbit, the palate, or the lower jaw, were then excluded, as were those cases that did not specify the exact location of the primary tumor.

## 3. Results

From 1968 to 2016, we found a total of 93 cases of ESFTs involving the maxilla or maxillary sinus published in the English medical literature (Table 1). Of these, 14 were further classified by the authors as being PNETS, mainly on account of their positivity for neuroendocrine markers. Of the 54 cases, in which patient's sex and age were specified, 32 were male (59.3%), 22 were female (just over 40%), and 36 patients were aged 25 or younger (66.6%). The slightly higher odds ratio for the male sex of 1.45, as well as the fact that the majority of cases describe patients in their first and second decade of life, seems congruent with the literature reported so far. All together 38 cases (40% of all the cases) reported positive immunohistochemical staining for MIC2/CD99. As from the year 1999, those cases including MIC2/CD99-status constitute 72% of total published cases and series. The transcription products EWS-FLI1, EWSR1, and EWS-ERG, on the other hand, were merely reported in 12 cases (only 13% of all cases), and 10 of these 12 cases were published since 2008. The vast majority of cases were identified as Ewing's sarcoma or PNETs on account of typical histological features, like Homer-Wright rosettes. 51 cases described patients' symptoms, probably leading to early diagnosis. Painless swelling was by far the most frequently reported symptom (31 cases, 60%), followed by congestion/obstruction (16 cases, 31%) and epistaxis (10 cases, 19.6%), while pain (5 cases) alongside proptosis with or without vision disorder (5 cases) seemed to occur seldom in less than 10% of patients. Mostly the onset of these symptoms occurred within the order of 3–6 months, thus leading to early diagnosis and therapeutic intervention accordingly. While only 4 cases failed to report therapy completely, 85 cases (91.4% of all cases) were treated with multimodality treatment, that is, the combination of at least local treatment (surgery and/or radiotherapy) plus systemic treatment. 11 cases reported the general term “chemoradiation” as the therapy implemented, without further specifying the exact cytostatic substances used, the radiation dose administered, or the sequence of these treatment modalities. The majority of 88 patients (95% of all cases) received some sort of local treatment via either radiotherapy, “chemoradiation,” or surgical excision (usually maxillectomy or lateral rhinotomy), and the latter was often combined with further adjuvant radiotherapy. All together 81 patients (87%) received systemic treatment in the form of chemotherapy (either neoadjuvant chemotherapy, adjuvant chemotherapy, or as “chemoradiation” not further specified), just over a third of them received this chemotherapy neoadjuvantly (29 patients, 35.8%). Follow-up was reported in the majority of 79 cases (only 14 were not reported). Of these, in total 68 patients (86%) remained disease free during the period of observation, only 9 deaths and 2 recurrences were reported all together.  Table 2 summarizes the follow-up and outcome data of the ESFT cases studied as a function of the implemented treatment modalities, respectively (only those cases including these specific data are represented; therefore, the case series by Biswas et al. [49] and Grevener et al. [50] were excluded). Maximum disease free periods were reported for radiochemotherapy with surgery (upon 16 years of follow-up [51]) and for radiotherapy without surgery (upon 23 years of follow-up [52]).

## 4. Discussion

### 4.1. Diagnosis and Differential Diagnosis of ESFTs

In the head and neck region, the differential diagnosis of small round-cell tumors includes lymphoma, malignant melanoma, rhabdomyosarcoma, olfactory neuroblastoma, undifferentiated carcinoma, and Ewing's sarcoma/pPNET. Melanoma, lymphoma and rhabdomyosarcoma can be identified with immunohistochemistry for S100, CD45, and desmin, respectively. In those cases of ESFTs which display focal positivity for S100 or desmin, additional immunohistochemical stains for melanoma markers (HMB45, Melan-A) and specific skeletal muscle markers (myogenin, myoD1) can be utilized to exclude melanoma and rhabdomyosarcoma. Carcinomas are generally diffusely positive for multiple keratins, whereas ESFTs typically stain focally for only one keratin marker [92]. Sometimes ESFTs can be positive for synaptophysin and other neuroendocrine markers (especially PNETs) but usually only stain focally. Almost 100% of ESFTs stain positively with CD99, while olfactory neuroblastomas do not [95]. The EWS-FLI1 fusion transcript, pathognomonic for ESFTs, does also not occur in neuroblastoma [96]. As outlined above, PNETs normally develop mainly in the central nervous system and soft tissue of children and young adults. When these tumors seldom occur outside the central nervous system, they are by definition termed peripheral primitive neuroectodermal tumor (pPNET). Peripheral primitive neuroectodermal tumors involving the maxilla are extremely rare disease entities [77]. In 1989, Coffin and Dehner described fewer than 10 reported cases of pPNETS involving the maxilla to have been published in English literature [97], while Mohindra et al. even spoke of less than 8 reported cases [83].

### 4.2. Prognostic Factors

Two-thirds of patients initially present with localized disease, which, when using multimodality treatment, is nowadays amenable to curation in approximately 70% of cases. However, patients presenting with primary metastatic disease (common sites being 10% in the lung, 10% in the bone/bone marrow, and 5% in combinations of lung and bone or other locations) generally have a poor chance of long-term survival, as this is the most important adverse prognostic factor. Therefore, complete staging, including at least CT of the chest and MRI with or without CT of the primary site, as well as PET scan and/or bone scan is regarded obligatory. In the staging of ESFTs, the combination of PET or PET/CT with conventional imaging has demonstrated sensitivity and specificity > 90% [98]. Other established adverse prognostic factors are increased tumor size, as unfavourable outcome has been shown for tumor volumes > 200 mL [39, 50], poor or no response to preoperative chemotherapy, as defined by >10% viability of residual tumor after neoadjuvant therapy [99, 100], elevated serum LDH level, axial localization, and older age (>15 years). The individual risk of relapse or disease progression however remains difficult to predict. While the EWS-ETS fusion type was shown to be prognostic within the retrospective de Alava et al. study [101], prospective evaluation of different EWS-FLI1 fusion architecture failed to reach statistical significance as independent prognostic markers [100]. When modern effective therapies are implemented, as reported from the EURO-EWING 99 study and the Children's Oncology Group study, patients with Ewing's sarcomas have similar outcomes, regardless of fusion subtype [100, 102].

### 4.3. Imaging and Staging

Classical Ewing's sarcoma, typically involving the long bones, radiologically presents as “onion skinning” periosteal reaction; although also described as a characteristic feature of neuroectodermal tumor lesions, this feature is definitely less frequently observed in the facial skeleton [78, 103]. Osteolytic lesions are not a pathognomonic radiologic feature of ESFTs, because other diseases like osteosarcoma, neuroblastoma, lymphosarcoma, osteomyelitis, and metastatic carcinoma can exhibit a similar image pattern [62]. In the skull, these tumors present as permeative, destructive lesions with large associated soft tissue components without calcifications, reflecting their aggressive nature. Radiologic features include “moth-eaten” permeative bony destruction, exuberant periosteal reaction (onion skin, sunburst, spiculated, hair on end), cortical erosion, and presence of an associated soft tissue mass. CT of Ewing's sarcoma of the PNS shows a diffusely enhancing soft tissue mass with bone destruction [57, 104–107]. Usually, no calcification is noted. Similar changes, however, might be seen in the other PNS tumors (squamous cell carcinoma, esthesioneuroblastoma, lymphoma, etc.). MRI findings of Ewing's sarcoma of the skull show an unusual pattern of reactive sclerosis [108]. MRI of Ewing's sarcoma typically shows the lesion as hypointense to isointense on T1W1 and hypointense to hyperintense on T2W1.

### 4.4. Local Therapy

Although there are no randomized studies comparing surgery and radiotherapy, data from retrospective analyses suggest better local control of early ESFTs achieved by surgery (with or without postoperative radiotherapy) than by radiotherapy alone [109]. Combined analysis from the CESS 81, CESS 86, and EICESS 92 trials even showed the rate of local failure after surgery to be significantly lower than after definitive radiotherapy without prior excision [109]. Therefore, if technically possible, complete surgical excision should be regarded the mainstay of local control. Only in those cases where complete surgical excision is not feasible (see below), should radiotherapy be applied alone at a dose of 45–60 Gy and then, however, preferably combined with systemic treatment. Mere surgical debulking procedures, aimed at tumor-downsizing without achieving complete resection, do not improve local control compared to definitive radiotherapy and should not be advocated as they are associated with additional morbidity. Data from the CESS and EICESS trials, showed that patients who had an intralesional resection followed by radiotherapy had the same local control rates as patients who received radiotherapy alone [109], thereby clearly negating the benefit of intralesional surgery. When local treatment modalities like surgery or radiotherapy are used without systemic chemotherapy, 5-year survival often remains <10%. Modern treatment protocols including systemic polychemotherapy regimens in multimodality trials render survival rates of up to 70% in localized and 20%–30% in metastatic disease, depending upon metastatic sites and burden [110, 111]. Grevener et al. have recently studied the outcome of Ewing sarcomas of the head and neck by analyzing the German Society for Pediatric Hematology and Oncology database between 1999 and 2009. This publication also included 7 cases of ESFTs involving the maxilla but found no difference in event free survival or overall survival when comparing the local treatment modalities: surgery, radiotherapy or combined surgery followed by adjuvant radiotherapy [50]. In our review of ESFTs confined to the maxilla and maxillary sinus, we found that a total of 88 patients (95% of all cases) received some sort of local treatment via either radiotherapy, “chemoradiation,” or surgical excision. Maxillectomy and lateral rhinotomy were the most frequently performed surgical techniques. Some of these patients then received further local control postoperatively by adjuvant radiotherapy. Several case series failed to discriminate between the exact modality of local therapy utilized, that is, merely reporting “radiotherapy or surgical resection” (with or without adjuvant radiotherapy). For this reason, it will not be possible to make a general recommendation concerning the optimal local treatment strategy based on this review data. The best treatment option for local tumor control in the maxilla or maxillary sinus will always need to be assessed individually, as each and every clinical case will present its unique challenges to the team of specialists involved.

Morbidity of local therapy is one of these key issues, which, when facing ESFTs in the maxilla and maxillary sinus region, will tremendously impact the choice of local treatment modality. Surgery of the middle face and skull is technically demanding. Radical tumor excision is often limited by the proximity of adjacent critical structures and complicated by the wish to preserve function and cosmesis. Extensive, mutilating facial surgery, leading to loss of physiognomy, functional defects, and cosmetic problems, will often provoke a multitude of further complications and severely compromise patients' quality of life, without therapeutic benefit in the long run. Major concerns related to surgical management, especially in children and adolescents, include deleterious effects on respiratory function, nutrition and deglutition, speech, and vision as well as overall facial appearance and cosmesis. On the other hand, the use of modern surgical techniques, implementing microvascular flaps, immediate reconstruction using PEEK (Polyetheretherketone) implants, titanium grid-plates, titanium-enforced Medpore-foils, obturator prosthesis, dental prosthesis, and other reconstructive surgical materials, often enable the surgeon to perform excellent functional and esthetical results nowadays [112]. Taking this into account, whenever complete surgical excision is threatening to provoke morbidity at a high cost for the patient, or even deemed to be technically impossible, then alternatively, considering ESFTs' pronounced radiosensitivity, radiotherapy may constitute a valid option for effective local tumor control. While radiation may seem less burdensome, it is certainly not without both early and late sequelae. Organ preservation does not automatically mean functional preservation. Radiotherapy in this region may also lead to serious adverse effects, such as mucositis and stomatitis, with consecutive loss of taste and appetite, as well as severe pain. Radiotherapy may harm the function of salivary and nasal glands, be detrimental to dental health status, and compromise nasal inspiration by injuring mucous membranes as well as causing a multitude of ocular disorders, like irritations and dryness, conjunctivitis, lens opacification, or even total blindness. Other late effects of radiotherapy in the facial region include auditory and vestibular defects. In the child and adolescent, long-term adverse effects on growth, behavioral problems, and cognitive deficits have also been reported. Modern intensity modulated radiotherapy (IMRT) protocols applied by radiooncology departments in specialized sarcoma centers can of course substantially reduce the risk of morbidity, by calculating and programming defined radiation tangents in order to minimize scattered radiation, otherwise harming surrounding structures. When IMRT is implemented accordingly, it can, for instance, avoid orbital sequelae as has been previously shown in the treatment of sinonasal tumors [113]. Radiation induced secondary tumors are another well-known late effect, especially an increased risk for osteosarcomas has frequently been described; these however tend to occur more often after treatment of ESFTs involving the extremities. The phenomenon of radiation induced secondary malignancy is clearly dose related; a significant increased risk for secondary tumors arises with administered radiation doses above 40 Gy [114, 115]. When radiotherapy has been administered as the primary local treatment modality (e.g., neoadjuvantly), subsequent surgery can lead to common complications, including wound infections, fistula formation, and the need for surgical revision, whereas flap survival does not seem to be negatively impacted by prior radiation [116]. Needless to say, it is imperative that local treatment modalities for ESFTs in the maxillary region be discussed multidisciplinary early on. Highly experienced maxillofacial surgeons, radiooncologists, and radiation-physicists, as well as medical oncologists and specialized nursing staff should be involved, thereby outweighing the advantages and risks of implementing a given therapeutic local strategy. As always in ESFT-management, but especially for ESFTs involving the confined maxillary region, the possibility of tumor downstaging by neoadjuvant systemic chemotherapy, alongside combating early micrometastatic disease, needs to be carefully evaluated and preferably utilized whenever possible.

### 4.5. Systemic Therapy

While high-dose chemotherapy followed by hematopoietic stem cell transplantation, although employed in some trial protocols in high-risk localized and metastatic ES [117], is still clearly considered investigational, the most active substances so far are regarded to be doxorubicin, cyclophosphamide, ifosfamide, vincristine, dactinomycin, and etoposide [39, 109, 118–120]. Combinations of these agents are initially employed neoadjuvantly during 3–6 cycles at 2-3-week intervals after histological diagnosis is confirmed by biopsy to downstage the tumor and increase the probability of achieving microscopically negative resection margins. Following surgical resection, further 6 to 10 cycles of adjuvant polychemotherapy have been shown to improve relapse free survival and overall survival [118, 119, 121–123]. Thereby, overall treatment duration usually reaches approximately 10–12 months. In the attempt of perfecting efficacy, a variety of regimens have been analyzed prospectively and retrospectively.

Data from the IESS-I and IESS-II trials could show that adjuvant chemotherapy with VACD (vincristine, dactinomycin, cyclophosphamide, and doxorubicin) leads to a significantly better 5-year relapse free survival (60% versus 24%) and overall survival (65% versus 28%) compared to VAC (vincristine, dactinomycin, and cyclophosphamide) when applied together with radiotherapy in localized nonmetastatic disease [119].

When ifosfamide and etoposide were added to this regimen (VACD-IE) in the Pediatric Oncology Group-Children's Cancer Group (POG-CCG) study, the 5-year event free survival rate (69% versus 54%) and the 5-year overall survival rate (72% versus 61%) were superior to VACD [124]. VACD-IE also showed lower cumulative incidences of local failure (11%) compared to VACD (30%), irrespective of the type of local control therapy [124]. In the INT 0091 study, patients with metastatic disease, however, did not profit from adding IE as there were no significant differences in 5-year event free survival or OS between VACD and VACD-IE [118]. In line with this data, the addition of etoposide to VAIA (EVAIA) was seemingly associated with a survival benefit (although not statistically significant) in the subgroup of patients without metastases in the EICESS-92 study [120]. The Euro-EWING 99 protocol for the treatment of localized disease is largely based on the same drug combinations used in the previous Cooperative Ewing's Sarcoma Study (CESS) and the European Intergroup Cooperative Ewing's Sarcoma Study (EICESS). Multiagent induction chemotherapy with six courses of VIDE (vincristine, ifosfamide, doxorubicin, and etoposide) followed by local treatment (surgery and/or RT) and HDT/SCT (high-dose chemotherapy/stem cell transplantation) were designed to evaluate efficacy and safety in patients with primary disseminated Ewing's sarcoma [99]. Of the 93 cases of ESFTs found in the maxilla or maxillary sinus region described in our review, only 7 failed to receive chemotherapy of some sort. The overwhelming majority was treated with polychemotherapy regimens including doxorubicin, cyclophosphamide, ifosfamide, vincristine, dactinomycin, and etoposide.

Complications and toxicities caused by chemotherapy are numerous and agent dependent. Besides the most common and often expected adverse effects, like fatigue, mucositis, and hemotoxicity, many cytostatic substances can cause agent-specific toxicity. For example, anthracyclines, including doxorubicin, may induce a dose-related cardiomyopathy leading to congestive heart failure. Alkylating agents, like cyclophosphamide and ifosfamide, are associated with infertility, especially male infertility, so that sperm cryopreservation should be offered to postpubertal boys and men wishing to father children, prior to the initiation of chemotherapy. Chemotherapy has also been associated with inducing secondary malignancies. There have been reports of 1%-2% increased rates of secondary leukemia following a sequence of chemotherapy protocols utilized for treating Ewing's sarcoma, and usually these malignancies occurred within 3 years of initial ESFT-diagnosis and therapy [118].

### 4.6. Follow-Up and Outcome

Upon completion of multimodality treatment for ESFTs of the maxilla or maxillary sinus, patients should be controlled clinically and radiologically in regular fixed intervals. In our review of the literature of ESFTs involving the maxilla and maxillary sinus, follow-up was reported in the majority of 79 cases (see Tables 1 and 2; only 14 cases failed to report follow-up at all). Of these, an overwhelming 68 Patients (86%) remained disease free during the period of observation, and only 9 deaths and 2 recurrences were reported all together. This can be accounted for mainly by the fact that all but one of the cases reported documented early ESFTs confined to the maxilla or maxillary sinus, thereby ruling out (or failing to mention) metastatic disease. The greater amount of tumor recurrences has however previously been described to take place within the first 2 years of follow-up [44]. Therefore, as some of the follow-up periods mentioned in this review are less than one year, some case series included median follow-up periods and others even failed to precisely outline deaths with respect to the exact primary tumor-site, and the actual numbers of true relapses and tumor-associated deaths may be indefinitely higher and so underrepresented by these figures. Taking these caveats into account, the overall prognosis for early ESFTs confined to the maxilla and maxillary sinus, as represented (see Table 2), seems nonetheless very optimistic. So bearing in mind, of course, that the collected data presented in this review are of selected cases only, these findings do stand in line with previous reports that tend to show a more favourable prognosis for ESFTs occurring in the head and neck region [50].

### 4.7. Recommendations for Daily Practice

In daily clinical practice, when facing a patient, especially pediatric patient or adolescent, presenting symptoms of acute or chronic unexplained facial swelling, a painless (or painful) mass of the upper jaw and nasal congestion or obstruction, as well as unexplained nose bleeding, we recommend further timely diagnostic procedures. These should at least include meticulous clinical examination of the jaw and oral cavity, endoscopy, biopsy for histology, and local imaging by X-ray or if necessary computed tomography. Once histologically confirmed, ESFTs should then be transferred to, or treated under close supervision of, a sarcoma center from an early stage. The necessity for further imaging (i.e., MRI of the head and neck, CT, and PET/CT), neoadjuvant treatment strategies, and precise preoperative surgical and/or radiotherapeutical planning is vital and needs to be evaluated by the involved MDT-specialists as soon as possible. This interdisciplinary decision-making process is just as important as the definitive skills and expertise of the sarcoma surgeon or radiooncologist involved in the next steps of treatment. In localized ESFTs of the maxilla and/or maxillary sinus, we recommend always preoperatively consulting a team of experienced maxillofacial surgeons alongside surgeons with profound experience in the field of plastic reconstructive surgery of the upper jaw and surgical dentistry. Radiooncologist and medical oncologists should be included in preoperative decision-making as well. Whenever feasible, neoadjuvant chemotherapy ideally within a clinical trial, containing a well-known and efficacious regimen like VIDE, should be administered and closely monitored by an experienced team of medical and/or pediatric oncologists. Therapeutic success (tumor-shrinkage) should be checked in regular 8–12-week intervals, radiologically as well as clinically if possible. The aim hereby should always be to boost the probability of complete R0-tumor-resection leading to microscopically free margins by maximum tumor-shrinkage, as well as destroying occult micrometastasis. If the risk of debilitating surgery causing serious personal morbidity to the patient, or the risk of incomplete tumor excision itself, is deemed high, then alternatively IMRT to the radiologically predefined tumor-bed should be evaluated by experienced radiooncologists. Decisions concerning the modality of adjuvant treatment following local tumor control should, when applicable, be made upon the defined histological regression grade, that is, pathologically reported vitality of the remaining tumor tissue (see above) excised after neoadjuvant systemic treatment. This treatment, once again, should ideally be performed under the umbrella of a randomized clinical trial; if not available, however, then at least it should be performed in close analogy to renown therapeutic protocols as, for instance, the EWING 99 study. Upon completion of multimodality treatment for ESFTs of the maxilla or maxillary sinus, patients should be controlled clinically and radiologically in regular fixed intervals. As most relapses have been reported to occur within the first two years after treatment [44], we would advocate close follow-up during the first 5 years by experienced clinicians at a sarcoma center. Endoscopic controls, when possible also performed behind a surgically placed prosthesis (ideally provisional and not definitely fixed during the first 3 years), as well as MRIs of the head and face and X-ray of the chest, should follow a strict 3–6 monthly schedule. After the first 5 years, it may be sufficient to perform follow-ups in greater intervals of 6–12 months, always, however, considering the patient's individual relapse-risk depending upon that mentioned above (resection margin status, radiotherapy dose, remission status following chemotherapy, etc.).

## 5. Conclusions

Although rare and potentially highly aggressive, ESFTs limited to the maxilla and maxillary sinus seem well manageable when utilizing modern multimodality treatment strategies. As described in the literature to date, outcome and prognosis of this specific entity seem more favourable compared to ESFTs occurring in the common primary sites located in the long bones or pelvis. This may in part be due to the fact, that patients presenting with ESFTs in the maxilla or maxillary sinus frequently experience symptoms like facial swelling, nasal congestion or even epistaxis at an early stage of tumor growth, ultimately leading to earlier diagnosis and successful treatment. Contrary to expectations, ESFTs occurring in the confined spatial proportions of the maxilla and maxillary sinus, often less amenable to complete surgical resection with clear tumor margins, may be equally successfully treated with a combination of radiotherapy and chemotherapy without surgery. Especially in those cases, in which the risk for morbidity by mutilating surgery seems high or complete surgical excision technically impossible, radiotherapy (combined with neoadjuvant and/or adjuvant chemotherapy) may prove to be similarly efficacious for achieving good local control without compromising long-term survival. In general we recommend consulting a sarcoma reference center early on in treatment planning, thereby ensuring for multidisciplinary expertise in favour of best clinical practice. As always, when available, patients with such rare tumor entities should definitely be enrolled in clinical trials or at least treated in accordance with modern trial protocols. So using well-established chemotherapy regimens neoadjuvantly and adjuvantly, in combination with surgery and/or radiotherapy for local control, will aim to achieve maximum possible treatment outcome.

## Figures and Tables

**Table 1:** Literature review 1968–2016.

Study	Year	Tumor/patient	Localisation	Symptoms	Therapy	Follow-up	IHC/Mol
Hunsuck [53]	1968	ES/m 33 y	Maxilla	Swelling	(1) RTX 3000 rads	18 mts: DF	n.s.
					(2) Subtotal maxillectomy		

Roca et al. [54] REV.	1968	ES m/3 y	Maxilla	Proptosis	RTX 4000 r + resection	11 y: DF	n.s.
		ES m/7 y	Maxilla	Swelling	Curettage, RTX 5000 r + C	5 y: DF	n.s.

Brownson and Cook [55]	1969	ES f/6 y	Maxilla	Facial swelling	(1) RTX (n.f.s.)	n.s.	n.s.
					(2) VC		n.s.

Fernandez et al. [56] REV.	1974	ES/2 Pat.	Maxilla	n.s.	Chemoradiation (n.f.s.)	n.s.	n.s.

Ferlito [57] REV.	1978	ES/m, 29 y	Maxilla	Congestion	RTX + Adr, V, Bl	10 mts: died	n.s.
		ES/m, 44 y	Maxilla	Swelling	Resection, RTX, Adr, V, CCNU	8 mts: DF	n.s.
		ES/m, 43 y	Maxilla	Epistaxis	RTX + Adr, V, CCNU	1 y: died	n.s.
		ES/m, 44 y	Maxilla	Epistaxis	RTX + Adr, V, CCNU	3 mts: DF	n.s.

Komray [58]	1979	ES/m, 15 y	Max sin	Epistaxis, exopht.	(1) RTX 6000 rads	1 y: DF	n.s.
					(2) VAdC		

Strong et al. [52] REV.	1979	ES/m, 3 y	Maxilla	n.s.	AMC + RTX 4000 rads	23 y: DF	n.s.
		ES/m, 7 y	Maxilla	n.s.	C + RTX 6500 rads	14 y: died	n.s.
		ES/m, 9 y	Maxilla	n.s.	CVAD + RTX 6000 rads	4 y: DF	n.s.

Pontius and Sebek [59]	1981	ES/m, 39 y	Max. sin.	Obstruct., lacrimat.	(1) Resection	2 y: DF	n.s.
					(2) RTX 7500 rads		

Hossfeld et al. [60]	1982	ES/n.s.	Maxilla	n.s.	Chemoradiation (n.f.s.)	n.s.	n.s.

Slootweg et al. [61]	1983	PNET/m, 10 y	Maxilla	Swelling	(1) Subtotal maxillectomy	3 y: DF	n.s.
					(2) RTX 50 Gy		

Bacchini et al. [62]	1986	ES/f, 7 y	Maxilla	n.s.	(1) Curettage, RTX 6000 rads	5 y 6 mts: DF	n.s.
					(2) Adj. CVAD		

Siegal et al. [42] REV.	1987	ES/m, 14 y	Maxilla	Swelling	VCD/VACD + RTX 4300 rads	10 y: DF	n.s.
		ES/m, 9 y	Maxilla	Swelling	VCD/VACD + RTX 6000 rads	8 y: DF	n.s.
		ES/f, 8 y	Maxilla	Swelling	VCD/VACD + RTX 6550 rads	7 y 5 mts: DF	n.s.
		ES/f, 15 y	Maxilla	Swelling	VCD/VACD + RTX 5500 rads	11 mts: DF	n.s.

Amin et al. [63]	1990	ES/f, 10 y	Maxilla	Obstruct., swelling	(1) Lateral rhinotomy	n.s.	n.s.
					(2) RTX, not performed		

Yeo et al. [64]	1991	PNET/f, 7 y	Maxilla	Swelling, pain	(1) Neoadj. VAC	9 y: DF	n.s.
					(2) Partial maxillectomy		
					(3) Adj. C + RTX 50 Gy		

Filiatrault et al. [65]	1993	PNET/f, 11 y	Max sin	Obstruct.	Chemoradiation (n.f.s.)	10 mts: SD	n.s.

Jones and McGill [66] REV.	1995	PNET/f, 13 y	Max sin	n.s.	Chemoradiation (n.f.s.)	2 y: DF	n.s.

Shah et al. [67]	1995	PNET/m, 42 y	Max sin	Swelling	(1) CAVCisPt, I, VP-16, E	9 mts: died	n.s.
					(2) RTX (n.f.s.)		

Ibarburen et al. [68]	1996	PNET/f, 20 mts	Maxilla	Swelling	n.s.	3 y: DF	n.s.

Fiorillo et al. [69]	1996	ES/m, 7 y	Maxilla	Swelling	CVAIE + RTX 6000 cGy	52 mts: DF	n.s.
		ES/f, 22 y	Maxilla	Swelling	CVAIE + RTX 6000 cGy	41 mts: DF	n.s.

Zheng et al. [70]	1998	ES/m, 16 y	Maxilla	Swelling	n.s.	n.s.	n.s.

Allam et al. [71] REV.	1999	ES/9 Pat.	Maxilla	n.s.	(1) RTX + VAdC + IEP/VAIA	3.4 y (mean)	n.s.
					(2) Surgery, n.s.		

Toda et al. [72]	1999	PNET/f, 57 y	Max. sin.	Obstruct.	(1) VAdC	3 mts: died	MIC2−
					(2) RTX 40 Gy		

Daw et al. [51]	2000	ES	Maxilla	n.s.	(1) Resection, RTX 3500 cGy	16 y 9 mts: DF	n.s.
					(2) Adj. CVAD, Carm		

Wexler et al. [73]	2003	ES	Max sin	Congestion, swelling	(1) Neoadj. VAdC + IE	3 y: DF	MIC2+, EWS-FLI1
					(2) Subtotal maxillectomy		
					(3) Adjuv. chemotherapy (n.f.s.)		

Kao et al. [74]	2002	PNET/m, 74 y	Maxilla	Swelling	CisPtEI	4 mts: died	CD99^+^

Alobid et al. [75]	2003	PNET/f, 23 y	Max. sin.	Obstruct.	(1) Lateral rhinotomy	59 mts: DF	CD99^+^
					(2) Adj. CVAD + RTX 60 Gy		

Harman et al. [76]	2003	ES/f, 40 y	Max sin	Obstruct.	(1) VAC	n.s.	CD99^+^
					(2) RTX 2000 cGy		

Windfuhr [44] REV.	2004	PNET/m, 7 y	Max. sin.	Exopht., vision loss	(1) Lateral rhinotomy	17 mts: DF	n.s.
					(2) Adj. VIDE/VACD		
					(3) RTX 45 Gy		

Howarth et al. [43]	2004	ES/m, 9 y	Maxilla	Swelling	(1) VIDE (inoperable)	n.s.	CD99^+^
					(2) RTX (n.f.s.)		

Sun et al. [77]	2007	PNET/f, 49 y	Maxilla	Swelling	(1) Total right maxillectomy	3 mts: DF	CD99^+^, EWS-FLI1
					(2) Radiotherapy 60 Gy		

Infante-Cossio et al. [78]	2005	ES/m, 17 y	Max. sin.	Swelling	(1) Neoadj. VCAIE	8 y: DF	CD99^+^
					(2) Complete resection		
					(3) Adj. chemotherapy (n.f.s.)		

Coskun et al. [79]	2005	ES/f, 16 y	Max sin	Swelling	(1) Surgery declined	1 y: DF	CD99^+^
					(2) RTX + CVAD/IE		

Varshney et al. [80]	2007	ES	Maxilla	Swelling	(1) Resection	n.s.	CD99^+^
					(2) Adj. VAC + RTX 2000 cGy		

Thariat et al. [81]	2008	ES/m, 26 y	Max. sin.	Swelling	(1) Neoadj. CD	11 y: local recurrence	t(11;22) EWSR1
					(2) Surgery		
					(3) Adj. AV		

Prasad et al. [82]	2008	ES/m, 18 y	Maxilla	Swelling	(1) Extended maxillectomy	1 y: DF	n.s.
					(2) Adj. chemotherapy (n.f.s.)		

Mohindra et al. [83]	2008	PNET/m, 5 y	Max. sin.	Swelling, exopht.	(1) VIDECAdr	n.s.	CD99^+^, EWS-FLI1
					(2) RTX 55.8 Gy		

Bornstein et al. [84]	2008	ES/f, 19 y	Max. sin.	Pain in teeth	(1) neoadj. VIDE (EU-Ew99)	n.s.	CD99^+^, EWS-FLI1
					(2) Hemimaxillectomy		
					(3) Adj. VAC + RTX 46 Gy		

Kawabata et al. [85] REV.	2008	ES, PNET/m, 12 y	Max sin	Swelling	(1) VDCIE	20 mts: DF	CD99^+^, EWS-ERG
					(2) RTX 45 Gy		

Ataergin et al. [86]	2009	ES/n.s.	Maxilla	n.s.	(1) Neoadj. IEAM	died	n.s.
					(2) Resection		
					(3) Adj. RTX (n.f.s.), VD IE AC		

Piloni et al. [87]	2009	ES/2 Pat.	n.s.	n.s.	n.s.	n.s.	n.s.

Gupta et al. [88]	2009	ES/m, 30 y	Maxilla	Swelling, congestion	(1) Excision	n.s.	CD99^+^
					(2) Adj. chemoradiation (n.f.s.)		

Hormozi et al. [89]	2010	PNET/f, 28 y	Maxilla	Diplopia	(1) Maxillectomy	6 mts: DF	CD99^+^
			Metastasis		(2) *γ*-knife (optic chiasma)		
			Parasellar		(3) Adj. VAI + RTX 80 Gy		

Dadhe et al. [90]	2010	ES/m, 12 y	Max. sin.	Swelling, pain	(1) Extended maxillectomy	1 y: DF	n.s.
					(2) Adj. chemotherapy (n.f.s.)		

Davido et al. [91]	2011	ES/m, 25 y	Max. sin.	Swelling, pain	(1) Neoadj. IDCisPt	5 y: DF	CD99^+^
					(2) Part maxillectomy		
					(3) Adj. IDCisPt		

Hafezi et al. [92] REV.	2011	ES/f, 69 y	Max. sin.	Obstruct., epistaxis	Chemoradiation (n.f.s.)	6 mts: DF	CD99^+^, EWS-FLI1
		ES/f, 45 y	Max. sin.	Obstruct., epistaxis	Chemoradiation (n.f.s.)	14 mts: died	CD99^+^, EWS-FLI1
		ES/m, 54 y	Maxilla	Obstruct., epistaxis	Chemoradiation (n.f.s.)	17 mts: died	CD99^+^ EWSR1
		ES/m, 25 y	Max. sin.	Obstruct., epistaxis	Surgery (n.f.s.)	26 mts: DF	CD99^+^ EWSR1
		ES/f, 13 y	Max. sin.	Obstruct., epistaxis	Surgery (n.f.s.)	4 mts: DF	CD99^+^ EWSR1
		ES/f, 15 y	Max. sin.	Obstruct., epistaxis	Chemoradiation (n.f.s.)	14 y: DF	CD99^+^ EWSR1

Yeshvanth et al. [93]	2012	ES/f, 29 y	Maxilla	Obstruct., epistaxis	(1) Neoadj. VACDex	1 y: DF	CD99^+^
					(2) Resection		

Kaler and Sheriff [94]	2013	ES/m, 22 y	Maxilla & orbit	Swelling, pain	(1) Craniofacial resection	2 y: local recurrence	CD99^+^
					(2) Chemoradiation (n.f.s.)		

Biswas et al. [49]	2014	ES/14 Pat.	Maxilla (sin.)	n.s.	(1) VDCIE	58 mts (median)	CD99^+^
					(2) RTX or surgery ± RTX 50–60 Gy		
					(3) Adj. chemotherapy (n.f.s.)		

Grevener et al. [50]	2016	ES/7 Pat.	Maxilla	n.s.	(1) VIDE	3.3 y (median)	n.s.
					(2) RTX or surgery ± RTX 44–54 Gy		

**Table 2:** ESFT, outcome as a function of treatment modality.

	Chemo TX, alone	Surgery, alone	Radio(chemo)TX (VAC, VCAD, VIDE) no surgery	Radio(chemo)TX (VAC, VCAD, VIDE) plus surgery	Treatment not specified
PNET	1 case [74]	—	6 cases	6 cases	1 case
Ewing	—	3 cases	23 cases	29 cases	3 cases
Disease free upon follow-up ≥ 1 year	—	1 case [92]	12 cases	15 cases	1 case [68]
Max. follow-up	4 months [74]	26 months [92]	23 years [52]	>16 years [51]	3 years [68]
Status upon max. follow-up	Dead	Disease free	Disease free	Disease free	Disease free

## Abbreviations

IHC: Immunohistochemistry
Mol: Molecular analysis
DF: Disease free
n.s.: Not specified
n.f.s.: Not further specified
RTX: Radiotherapy
VC: Vincristine and cyclophosphamide
Adr: Adriamycin
V: Vincristine
Bl: Bleomycin
CCNU: Chloroethylcyclohexylnitrosourea
VAdC: Vincristine, Adriamycin, and cyclophosphamide
AMC: Amethopterin and Cyclophosphamide
C: Cyclophosphamide
CVAD: Cyclophosphamide, vincristine, actinomycin, and doxorubicin
VCD: Vincristine, cyclophosphamide, and dactinomycin
VAC: Vincristine, actinomycin, and Cyclophosphamide
VACD: Vincristine, Adriamycin, cyclophosphamide, and dactinomycin
CAVCisPt: Cyclophosphamide, Adriamycin, vincristine, and cisplatin
I: Ifosfamide
E: Etoposide
VAIA: Vincristine, Adriamycin, ifosfamide, and actinomycin
CVAIE: Cyclophosphamide, Vincristine, actinomycin, ifosfamide, and etoposide
IEP: Ifosfamide, etoposide, and cisplatin
Carm: Carmustine
IE: Ifosfamide and etoposide
CisPtEI: Cisplatin, etoposide, and ifosfamide
VIDE: Vincristine, ifosfamide, doxorubicin, and etoposide
VCAIE: Vincristine, cyclophosphamide, actinomycin, ifosfamide, and etoposide
VIDECAdr: Vincristine, ifosfamide, dactinomycin, etoposide, cyclophosphamide, and Adriamycin
VDCIE: Vincristine, doxorubicin, cyclophosphamide, ifosfamide, and etoposide
IEAM: Ifosfamide, etoposide, actinomycin, and melphalan
VD IE AC: Vincristine, dactinomycin, ifosfamide, etoposide, doxorubicine, and cyclophosphamide
VAI: Vincristin, actinomycin, and ifosfamide
IDCisPt: Ifosfamide, doxorubicin, and cisplatin
CD: Cyclophosphamide and doxorubicine
AV: Actinomycin and vincristine
VACDex: Vincristine, Adriamycin, cyclophosphamide, and dexamethasone.
